# Functional Characterization of S100A8 and S100A9 in Altering Monolayer Permeability of Human Umbilical Endothelial Cells

**DOI:** 10.1371/journal.pone.0090472

**Published:** 2014-03-03

**Authors:** Liqun Wang, Haihua Luo, Xiaohuan Chen, Yong Jiang, Qiaobing Huang

**Affiliations:** 1 Key Lab for Shock and Microcirculation Research of Guangdong, Department of Pathophysiology, Southern Medical University, Guangzhou, P. R. China; 2 Key Laboratory for Functional Proteomics of Guangdong Province, Department of Pathophysiology, Southern Medical University, Guangzhou, P. R. China; University of Miami, United States of America

## Abstract

S100A8, S100A9 and S100A8/A9 complexes have been known as important endogenous damage-associated molecular pattern (DAMP) proteins. But the pathophysiological roles of S100A8, S100A9 and S100A8/A9 in cardiovascular diseases are incompletely explained. In this present study, the effects of homo S100A8, S100A9 and their hetero-complex S100A8/A9 on endothelial barrier function were tested respectively in cultured human umbilical venous endothelial cells (HUVECs). The involvement of TLR4 and RAGE were observed by using inhibitor of TLR4 and blocking antibody of RAGE. The clarification of different MAPK subtypes in S100A8/A9-induced endothelial response was implemented by using specific inhibitors. The calcium-dependency was detected in the absence of Ca^2+^ or in the presence of gradient-dose Ca^2+^. The results showed that S100A8, S100A9 and S100A8/A9 could induce F-actin and ZO-1 disorganization in HUVECs and evoked the increases of HUVEC monolayer permeability in a dose- and time-dependent manner. The effects of S100A8, S100A9 and S100A8/A9 on endothelial barrier function depended on the activation of p38 and ERK1/2 signal pathways through receptors TLR4 and RAGE. Most importantly, we revealed the preference of S100A8 on TLR4 and S100A9 on RAGE in HUVECs. The results also showed the calcium dependency in S100A8- and S100A9-evoked endothelial response, indicating that calcium dependency on formation of S100A8 or A9 dimmers might be the prerequisite for this endothelial functional alteration.

## Introduction

The calcium-binding proteins S100A8 and S100A9 are pivotal mediators of inflammatory and protective anti-infection responses for the mammalian host [1]–[4]. S100A8 and S100A9 form S100A8/A9 heterodimers (calprotectin) and these proteins and complex have been identified as important endogenous damage-associated molecular pattern (DAMP) proteins.

S100A8 or S100A9 shows two calcium-binding sites (EF hands) per protein chain, one of high and one of low affinity for Ca^2+^ ions. The purified fraction of the S100A8/A9 was found to contain monomers and dimmers. S100A8 and S100A9 are known to form dimmers with themselves, and to form noncovalently linked protein complexes with each other in a Ca^2+^-dependent manner [5], [6]. The S100A8/A9 complex assembly is a Ca^2+^-regulated process.

There is a discrepancy in the effectiveness of different form of S100A8/A9 complex in pro-inflammatory process. S100A8 and S100A9 are known to form heterodimers predominantly under physiological conditions [7]. Ehlermann P et al. reported that heterodimeric S100A8/A9 was much more effective than homodimers of S100A8, or S100A9 in enhancing the expression of IL-6, ICAM-1, VCAM-1 and MCP1 in advanced glycation end products (AGE)-albumin pretreated HUVECs [8]. Schelbergen R et al.'s report showed that catabolic enzymes MMP-1, MMP-9, and MMP-13 and proinflammatory cytokine IL-6 were up-regulated by S100A8 and S100A9, but not by the S100A8/A9 heterodimer in culture human cartilage explants [9]. It is showed that glucose-mediated endothelial cell cytotoxicity was reduced via knockdown of S100A8, but not S100A9 [10]. These evidences suggest that S100A8 and S100A9 might have functions that are dependent, or independent, on hetero-complex formation and these functions could be regulated in part by different mechanisms [11].

Despite functioning as a proinflammatory mediator, the pathophysiological roles of S100A8, S100A9, and S100A8/A9 complexes in cardiovascular disease are incompletely defined [12]. S100A8 and S100A9 are abundantly expressed in neutrophils, monocytes, and in some secretory epithelia. Activated cells release S100A8 and S100A9 into the extracellular compartment to promote the adhesion of neutrophils to endothelium, to act as chemotactants on monocytes, and to enhance the uptake of LDL cholesterol by macrophages [13], [14].

The responses in many inflammatory disorders trigger the mass release of S100A8, S100A9 and S100A8/A9 from phagocytes [15], [16]. In inflamed tissues, the MRP-8/14 complex is deposited onto the endothelium of venules associated with extravasating leukocytes [17]. The changes of circulating levels of S100A8/A9 were associated with endothelial dysfunction [18]. A long-term challenge of S100A8/A9 complexes induces inflammatory and pro-thrombotic response in endothelial cells in vitro through enhancement of relative gene expression. It has been revealed that S100A9 disrupts endothelial cell integrity and decreases transendothelial resistance by inducing the expression of pro-inflammatory mediators and adhesion molecules in culture microvascular endothelial cells [19].

The released S100A8, S100A9, and complex S100A8/A9 induce their cellular effects by binding with Toll-like receptor-4 (TLR-4) [9], the receptor for AGE (RAGE) [8], and carboxylated glycans [17], [20] in target cells. The expressions of TLR4 and RAGE in endothelial cells are preonunced and inducible by inflammatory stimulation [21], [22]. Evidences have suggested that S100A8, S100A9 and S100A8/A9 might have different dependencies on TLR-4, RAGE, and even carboxylated glycans, depends on the species and the cell types used [17]. The preferential receptors employed may depend on the pathological settings, cell types involved and local ligand concentrations [23].

The binding of S100A8/A9 and their receptors will eli**c**it a series of signal transductions. Since mitogen-activated protein kinases (MAPKs) are the major downstream pathway for both TLR4 and RAGE, it is logical to consider the roles of MAPKs in S100A8 and/or A9-induced responses. Evidences have approved that S100A8, A9 promote their biological effects in a MAPK-dependent way [24].

It is still not clear whether extracellular S100A8 and/or A9 could directly affect the endothelial barrier function in an instant and early reaction. The different participations of monomers or homodimers of S100A8, S100A9 and heterocomplex S100A8/A9 in endothelial response are still blurred. We hypothesize that S100A8, S100A9, and S100A8/A9 bind to TLR4 and RAGE on endothelial cells in a calcium-dependent way, and then trigger the activation of MAPK pathways, resulting in the disorganization of cytoskeleton and redistribution of intercellular junctional proteins, ending up in endothelial barrier disruption and increase of monolayer permeability. In this study, we tested the effects of homo S100A8, S100A9 and hetero-complex S100A8/A9, respectively, on endothelial barrier function in cultured HUVECs. The calcium-dependency was detected in the absent of Ca^2+^ or in the presence of gradient-dose Ca^2+^. The involvement of TLR4 and RAGE were observed by using inhibitor of TLR4 and blocking antibody of RAGE. The clarification of different MAPK subtypes in S100A8 and/or A9-induced endothelial response was implemented by using specific inhibitors.

## Materials and Methods

### Chemicals and reagents

Primary human umbilical endothelial cell (HUVECs) line was from Sciencell. DMEM/F12 medium, fetal bovine serum (FBS), trypsin, glutamine, penicillin, and streptomycin were all from Gibco BRL (Grand Island, NY, USA). Antibodies against p-p38 (Cat. 4511), total p38 (Cat. 9212), p-ERK1/2 (Cat. 4370), and ERK1/2 (Cat. 4695) came from Cell Signaling Technology (Beverly, MA, USA). Antibody recognizing p-JNK (Cat. sc-6254) and total JNK (Cat. sc-7345), and primary antibody recognizing tight junction scaffolding protein ZO-1 were from Santa Cruz Biotechnology ((Cat. sc-10804) Santa Cruz, CA, USA). Secondary antibody was from Boisynthesis ((Cat. bse-0295G, bse-0296G), Beijing, China). MEK1 inhibitor PD98059 (Cat. P215), p38 inhibitor SB203580 (Cat. S8307), and JNK inhibitor SP100625 (Cat. S5567), FITC-anti-mouse IgG second antibody (Cat. F0382), and rhodamine-phalloidin (Cat. 19083) were acquired from Sigma (St. Louis, MO, USA). Specific TLR4 inhibitor TAK242 was from InvivoGen ((Cat. TLRl-cli95), San Diego, CA, USA). Human RAGE blocking antibody was obtained from R&D systems ((Cat. MAB11451), Minneapolis, MN) and at 10 µg/mL, this antibody will block >90% of the binding. Chemicals were purchased from Sigma (St. Louis, MO, USA) unless otherwise indicated.

### Preparation of S100A8, S100A9, S100A8/A9

Human recombinant S100A8, S100A9, and S100A8/A9 heterodimer complex were expressed and purified according to previous descriptions [7], [25] with some modifications. The S100A8 and S100A9 cDNAs were amplified from cDNA library of human pulmonary artery. The primers S100A8-5′ (5′-TCACATATGTTGACCGAGCTGGAGAAA-3′) and S100A8-3′ (5′-ATAGGTACCCTCTTTGTGGCTTTCTTCATGG-3′) were used to amplify the human S100A8 gene for the pET14b-MCS-hS100A8 expression vector, and the primers S100A9-5′ (5′-AGTATCACATATGACTTGCAAAATGTCGCAGCTGGAAC-3′) and S100A9-3′ (5′-TATATAGGTACCGGGGGTGCCCTCCCCGAG -3′) were used to amplify the human S100A9 gene for the pET14b-MCS-hS100A9 expression vector. A NdeI restriction site was engineered into the 5′-primers, and a Kpn I restriction site was engineered into the 3′-primers for proper insertion of the gene into the pET14b-MCS expression vector. The PCR products were sequenced with double deoxidution termination method. Over expression of the S100A8, A9 was obtained through a combination of pET14b-MCS vector and the Escherichia coli DH5α and BL21 (DE3) cell line. And the proteins were purified to homogeneity by using Ni^2+^-NTA- agarose column and then detected by SDS-PAGE electrophoresis and quantified by Brandford method. The S100A8/A9 heterodimerization was performed by mixing S100A8 and S100A9 proteins in an equimolar solution containing 10 mM Tris-HCl, pH 7.4, 0.1% sodium cholate, 1 mM EDTA and 1 mM β-mercaptoethanol. Then CaCl_2_ from a stock solution was added to a final concentration of 2 mM followed by 10 min incubation under ambient temperature. The efficiency of this procedure was assessed using a specific ELISA kit (Bühlmann Laboratories AG, Switzerland) for the detection of S100A8/S100A9 heterodimer [8]. Endotoxin contaminations were excluded by the Limulus amebocyte lysate assay with a minimum LPS sensitivity of 0.125 EU/mL (BioWhittaker) and in blocking experiments using polymyxin B (10 µg/ml) (Sigma).

### Culture and Stimulations of HUVECs

HUVECs were maintained in DMEM/F12 containing 10% FBS at 37°C in a humidified atmosphere with 5% CO_2_. In all experiments, HUVECs were grown to 90% confluence and starved of serum for 12 hours before being stimulated with S100A8, S100A9 or S100A8/A9 at the indicated doses and for the indicated times. DMEM/F12 medium was used as vehicle control [8]. For the detection of signal pathways of S100A8 and/or A9, specific inhibitor of TLR4, blocking antibody of RAGE, or inhibitor of relative MAPK was added 60 min before relevant S100A8, A9 applications, respectively. In calcium-dependent experiment, S100A8, S100A9 or S100A8/A9 was applied in normal calcium concentration, gradient-content calcium or calcium-free conditions. The calcium-free medium is standard PBS containing NaCl 8.0 g/L, KCl 0.2 g/L, Na_2_HPO_4_•2H_2_O 1.56 g/L, and KH_2_PO_4_ 0.2 g/L.

### Transendothelial electrical resistance (TER)

Transendothelial electrical resistance (TER) of HUVEC monolayer was determined using STX2 electrode and EVOM^2^ meter according to the instruction manual of manufacture (World Precision Instruments, Sarasota, FL, USA) [26], [27]. HUVECs were seeded with number of 1×10^5^/cm^2^ on fibronectin-coated, 6.5 mm Transwell filters (0.4 µm pore size) and were used until full confluence. Resistance values of multiple Transwell inserts of an experimental group were measured sequentially and the mean was expressed in the common unit (Ω cm^2^) after subtraction of the value of a blank cell-free filter.

### Immunoblotting

Total cellular extracts were prepared by lysis and sonication of the cells in lysis buffer (20 mmol/L Tris pH 7.4, 2.5 mmol/L EDTA, 1% Triton X-100, 1% deoxycholic acid, 0.1% SDS, 100 mmol/L NaCl, 10 mmol/L NaF, 1 mmol/L Na_3_VO_4_) with protease and phosphatase inhibitors. Samples were subjected to SDS-PAGE, and proteins were transferred to polyvinylidene fluoride (PVDF) membranes. Blots were blocked with 5% bovine serum albumin in TBS containing 0.5% Tween 20 (TBS-T) for 2 h and then incubated with an 1∶1000 dilution of primary antibody for p-p38, p-ERK1/2, p-JNK or total p38, ERK1/2, JNK overnight at 4°C on a rocker. After three washes for 10 min each with TBS-T, the blots were incubated with a 1∶5000 dilution of HRP-conjugated species-specific respective secondary antibody for 1 h at room temperature. After washing three times for 10 min each with TBS-T, protein bands were visualized by chemiluminescence and then densitometric analysis was done by using Kodak IS4000R Imaging Station.

### Immunofluorescent test

The distribution of tight junction protein ZO-1 and cytoskeleton F-actin in HUVECs were observed with immunofluorescent test. S100A8, S100A9 or S100A8/A9 in 2 µg/mL was added respectively, in culture medium for 0, 10, 30, 60 and 120 min. Subsequently, the medium was removed and the cells were washed with PBS, permeabilized with 4% formaldehyde and 0.5% Triton X-100 for 30 min in 4°C. Cells were washed in PBS twice, blocked in 5% BSA for 1 h, and incubated with ZO-1 antibody (1∶500) at 4°C overnight. After a thorough wash in PBS, the cells were stained with a FITC-conjugated secondary antibody (1∶2000) and conjugated rhodamine-phalloidin (2 U/mL) for 1 h. Cells were then imaged with a Zeiss LSM780 laser confocal scanning microscope (Zeiss, Germany).

### Statistical analysis

Data were normalized to control values and are reported as percentage of the baseline values (mean ± s.d.) from at least three independent experiments. Results were analyzed by one-way ANOVA followed by post hoc comparison. The level of significance was set at P<0.05.

## Results

### Effect of S100A8 and/or A9 on endothelial monolayer permeability

**Figure 1:**
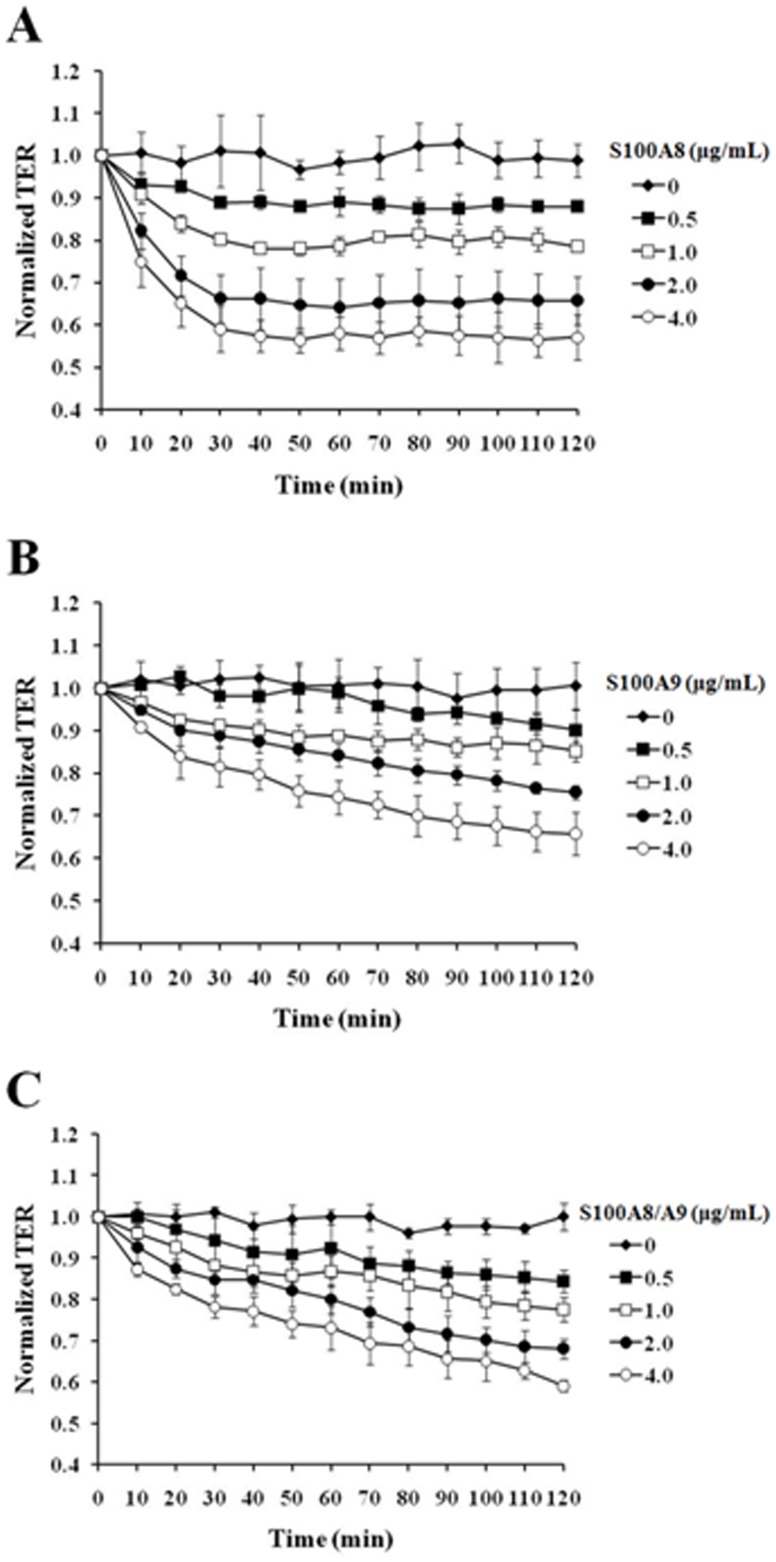
S100A8, S100A9 and S100A8/A9 induced a concentration- and time-dependent increase in HUVEC permeability. Filter-grown HUVEC monolayers were stimulated for 120 min with S100A8 (0, 0.5, 1.0, 2.0 and 4.0 µg/mL) (**A**), S100A9 (0, 0.5, 1.0, 2.0 and 4.0 µg/mL) (**B**) and S100A8/A9 (0, 0.5, 1.0, 2.0 and 4.0 µg/mL) (**C**) respectively. The TER was measured every 10 min. All data are presented as mean ± s.d. of four independent experiments. The statistical details is showed in Fig. S1.

The influence of S100A8, S100A9 or S100A8/A9 on HUVEC monolayer permeability was examined by detecting the TER of EC monolayer. S100A8, S100A9 or S100A8/A9 with concentrations of 0, 0.5, 1.0, 2.0, 4.0 µg/mL were applied, respectively, to filter-grown HUVECs in passages of 3∼5. The monolayer TER was measured for 2 h in a 10 min interval. Both S100A8 and S100A9 evoked the decreases of TER in a dose- and time-dependent manner. S100A8-induced barrier disruption demonstrated a sharper decline and reached the bottom in 30 min, then sustained for over 2 h (Fig. 1A), while S100A9 showed a more gradual decrease in TER in 2 h (Fig. 1B). The hetero-complex of S100A8/S100A9 also induced the decrease of TER in less than 30 min (Fig. 1C). The statistical details is showed in Fig. S1.

### S100A8, A9 induced F-actin and ZO-1 disorganization in HUVECs

**Figure 2:**
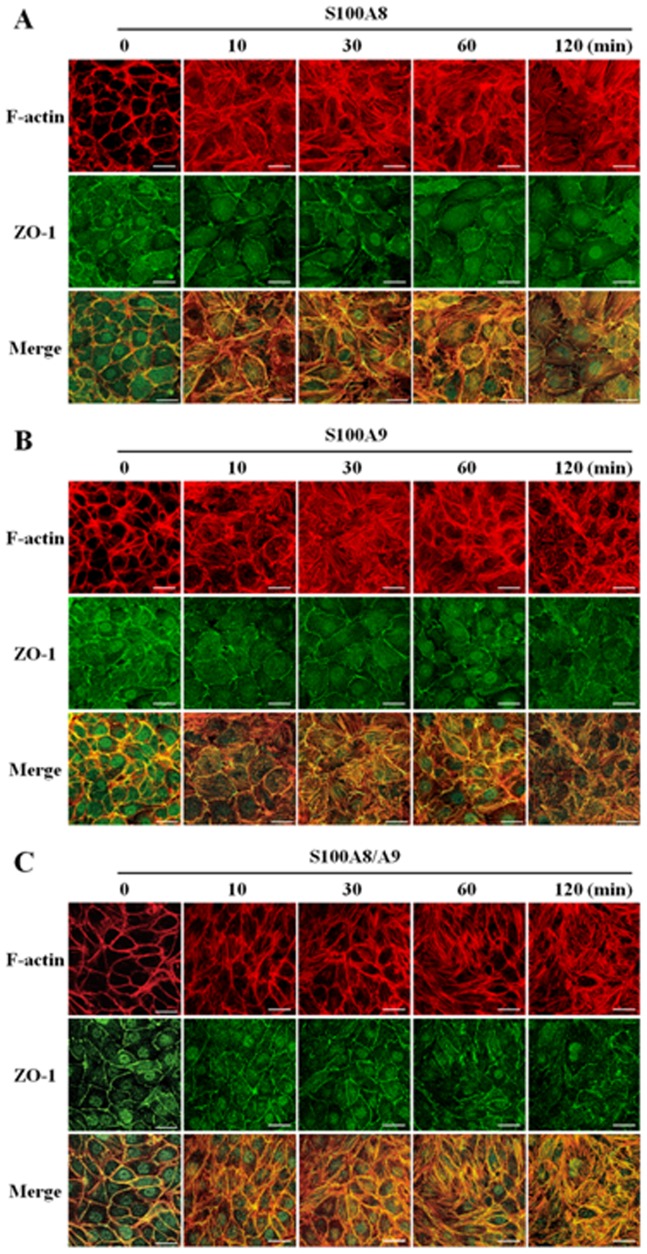
S100A8, S100A9 and S100A8/A9 induced stress fiber formation and disrupted ZO-1. HUVECs grown on Petri dishes were incubated with S100A8 (2.0 µg/mL) (**A**), S100A9 (2.0 µg/mL) (**B**) and S100A8/A9 (2.0 µg/mL) (**C**) for 0, 10, 30, 60 and 120 min respectively. Cells were fixed, and double immunofluorent staining was performed according to the protocol described in Materials and Methods. Cells were probed with rodamine-conjugated phalloidin to detect actin filaments (Red) and with ZO-1 antibody to label tight junctions (Green). Scale bar, 30 µm. Results are representative of three independent experiments.

Smooth and continuous staining for both F-actin and ZO-1 along with the intercellular borders of adjacent endothelial cells was seen in untreated HUVECs. The exposure of S100A8, S100A9 or S100A8/A9 in 2.0 µg/mL to HUVECs caused a noticeable alteration of ZO-1 spreading at cellular border, displaying discontinuity and serration in the location of per se sharp lining without stimulation. This change was accompanied by the polymerization of F-actin and the formation of stress fiber, resulting in the appearance of intercellular gaps. These changes became more obvious as the exposure time extended from 10 to 120 min (Fig. 2).

### Involvement of MAPKs in S100A8 and/or A9-induced endothelial barrier disruption

**Figure 3:**
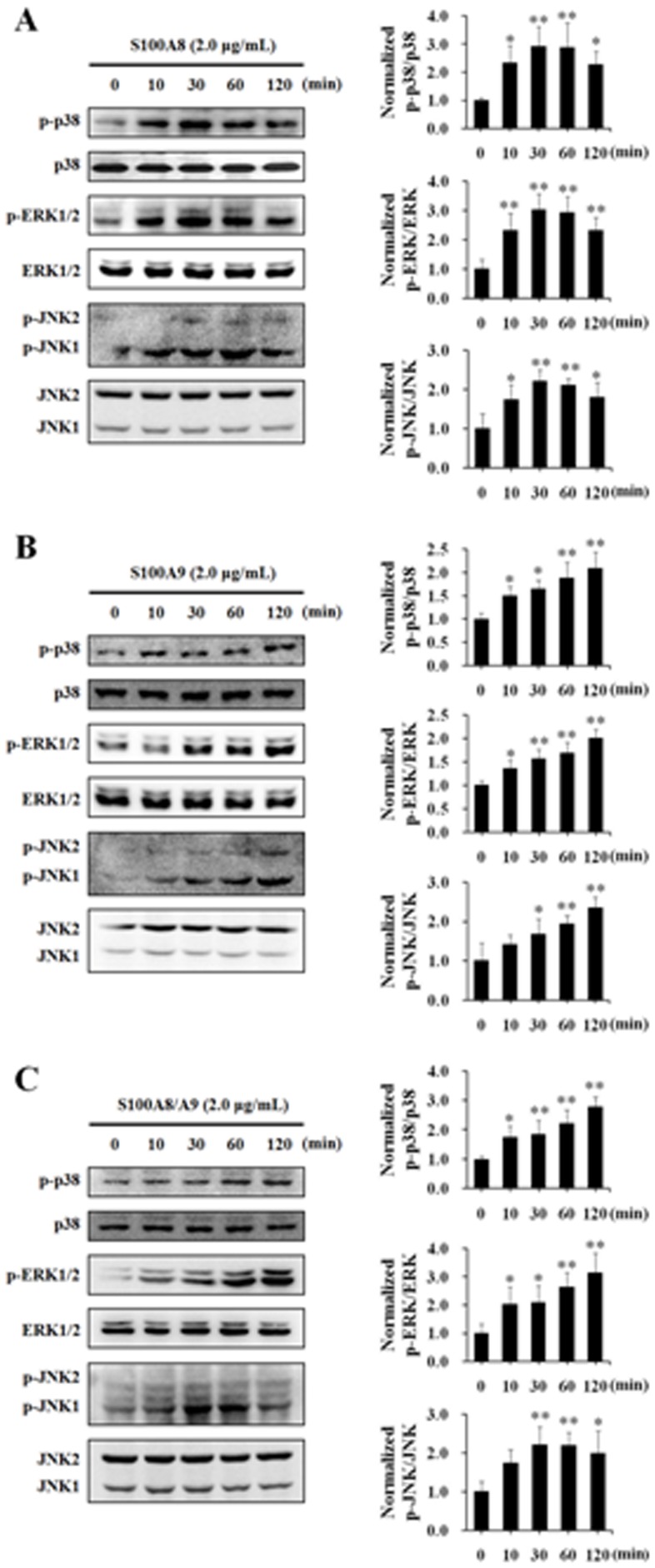
S100A8, S100A9 and S100A8/A9 induced the phosphorylation and activation of MAPKs. HUVECs were stimulated with S100A8 (2.0 µg/mL) (**A**), S100A9 (2.0 µg/mL) (**B**) and S100A8/A9 (2.0 µg/mL) (**C**) for 0, 10, 30, 60 and 120 min respectively. Phosphorylation of p38 (p-p38), ERK1/2 (p-ERK1/2) and JNK (p-JNK) were assessed by Western blotting with specific antibodies as described in Materials and Methods. The ration of immunointensity between the phosphorylation of MAPKs (p-p38, p-ERK and p-JNK) and total MAPKs (p38, ERK and JNK) were calculated. The results are expressed in mean ± s.d. from three independent experiments. ^*^P<0.05 vs. Control; ^**^P<0.01 vs. Control.

To explore the signal mechanism of S100A8 and/or A9-induced endothelial dysfunction, the phosphorylation of three MAPK subtypes, p38, ERK1/2, and JNK was determined by Western blot after HUVECs were treated with S100A8, S100A9 or S100A8/A9 in a dose of 2.0 µg/mL for 10, 30, 60 and 120 min, respectively. The results displayed that the application of S100A8, S100A9, or S100A8/A9 remarkably induced the phosphorylation of p38, ERK1/2, and JNK respectively. S100A8 sparked the phosphorylation as early as in 10 min and the phosphorylation of MAPKs reached the peak in 30 min and began to decline in 2 h (Fig. 3A). S100A9 increased the phosphorylation of p38, ERK1/2, and JNK gradually in the experimental period of 120 min (Fig. 3B). The administration of S100A8/A9 also induced the phosphorylation of these MAPK subtypes in 10–30 min (Fig. 3C). The time-dependent MAPK phosphorylation induced by S100A8 and S100A9 seemed to match the pattern of time-dependent decrease of TER in HUVECs, respectively.

**Figure 4:**
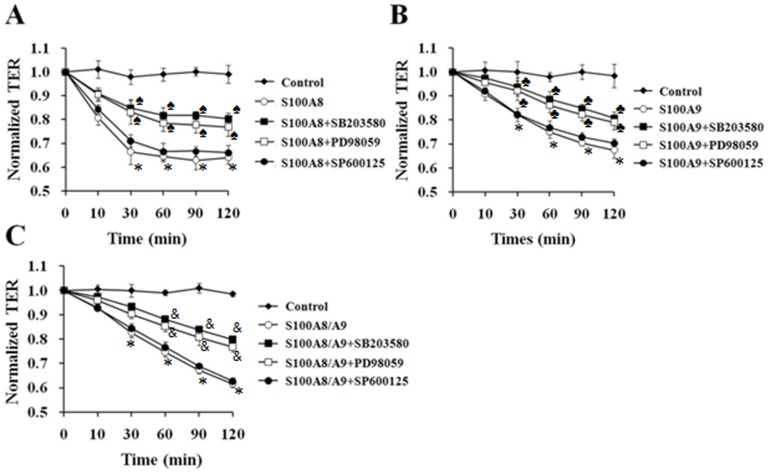
S100A8, S100A9 and S100A8/A9 induced increase in HUVEC permeability by a p38 and ERK1/2 dependent mechanism. HUVECs were stimulated with S100A8 (2.0 µg/mL) (**A**), S100A9 (2.0 µg/mL) (**B**) or S100A8/A9 (2.0 µg/mL) (**C**) for 120 min, with or without 60 min pre-incubated with SB203580, PD98059 and SP600125 which were used as standard inhibitor for p38, ERK1/2 and JNK, respectively. Then the TER was measured. All data are presented as mean ± s.d. of four independent experiments. ^*^P<0.05 vs. Control, ^♠^P<0.05 vs. S100A8, ^♣^P<0.05 vs. S100A9, ^&^P<0.05 vs. S100A8/A9.

**Figure 5:**
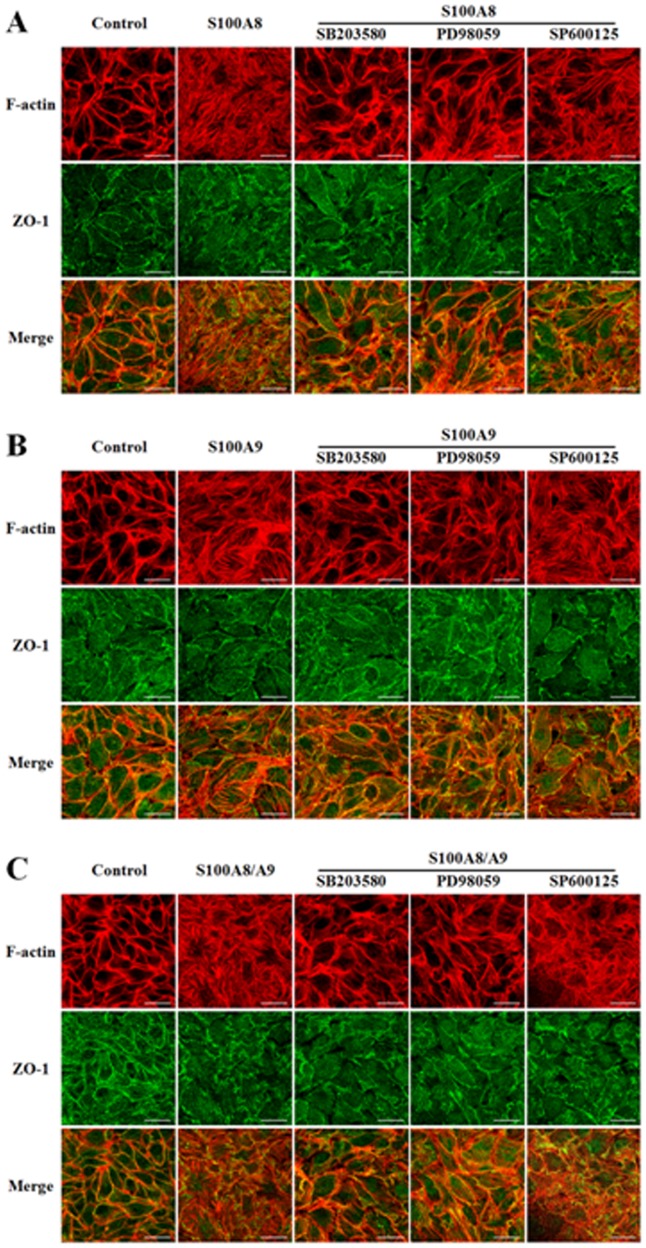
S100A8, S100A9 and S100A8/A9 induced stress fiber formation and disrupted ZO-1 by a p38 and ERK1/2 dependent mechanism. HUVECs were stimulated with S100A8 (2.0 µg/mL) (**A**), S100A9 (2.0 µg/mL) (**B**) or S100A8/A9 (2.0 µg/mL) (**C**) for 120 min, with or without 60 min pre-incubated with SB203580 (10 µM), PD98059 (50 µM) and SP600125 (10 µM), which were used as standard inhibitor for p38, ERK1/2 and JNK, respectively. Cells were fixed and double immunofluorent staining was performed according to the protocol described in Materials and Methods. Cells were probed with rodamine-conjugated phalloidin to detect actin filaments (Red) and with ZO-1 antibody to label tight junctions (Green). Scale bar, 30 µm. Results are representative of three independent experiments.

To further clarify the involvement of MAPKs in S100A8 and/or A9-induced endothelial barrier disruption, HUVECs were pretreated with inhibitor of p38 SB203580 (10 µM), inhibitor of ERK1/2 PD98059 (50 µM), and inhibitor of JNK SP600125 (10 µM) for 60 min, respectively, before the application of S100A8, A9. TER were then measured in a period of 120 min. The results revealed that the inhibition of p38 or ERK1/2 activation significantly attenuated the increase of endothelial monolayer permeability induced by S100A8, S100A9 or S100A8/A9. The inhibition of JNK phosphorylation showed no influence on S100A8 and/or A9-induced endothelial barrier dysfunction (Fig. 4). These data declare that p38 and ERK1/2, but not JNK, are involved in S100A8 and/or A9-induced endothelial hyper-permeability response. The morphological changes of endothelial cytoskeleton and tight junction protein induced by S100A8 and/or A9 were also abolished by inhibiting the activation of p38 and ERK1/2 while the formation of stress fiber and the serrating discontinuity of ZO-1 were attenuated in SB203580 or PD98059-pretreated HUVECs. The inhibition of JNK showed no effect on the disorganization of F-actin and ZO-1 induced by S100A8 and/or A9 (Fig. 5). The effect of JNK activation in S100A8 and/or A9-induced endothelial response might relate to other pathological process, such as the synthesis and release of cytokines and adhension molecules.

The possibility of contaminated LPS-induced endothelial hyperpermeability and MAPK activation was excluded by heat-inactivation of solutions. S100A8, S100A9 and S100A8/A9 lost their activities while being heat-inactivated at 80°C for 30 minutes. LPS activity was not changed at that temperature [9], [25]. The results demonstrated that inactivated S100A8, S100A9, or S100A8/S100A9 had no influence on HUVEC TER and phosphorylation of ERK1/2, excluding the possibility of LPS contamination (Fig. S2).

### The receptors for S100A8 and S100A9 in endothelial responses

**Figure 6:**
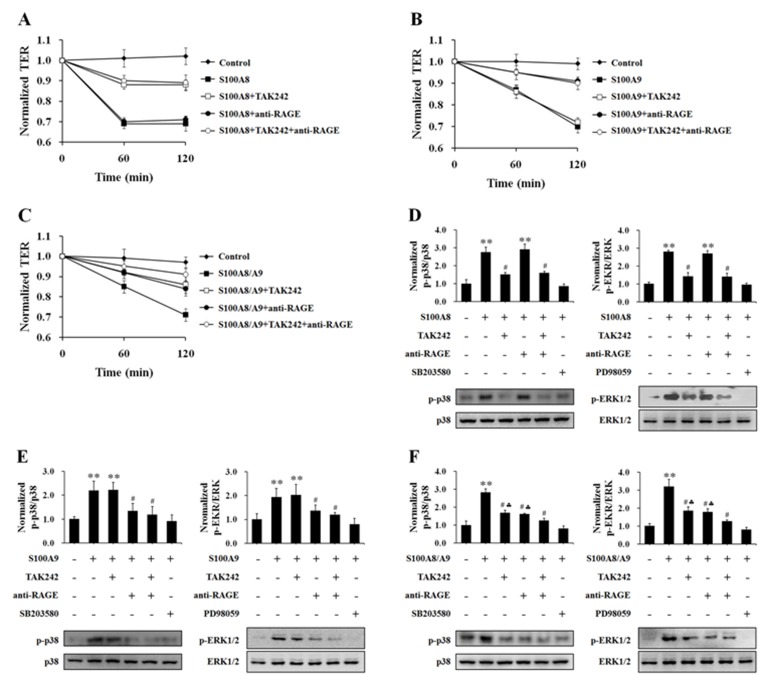
The effects of blocking TLR4 and RAGE on S100A8, S100A9 and S100A8/A9 stimulation of HUVECs. HUVECs were stimulated with S100A8 (2.0 µg/mL) (**A, D**), S100A9 (2.0 µg/mL) (**B, E**) and S100A8/A9 (2.0 µg/mL) (**C, F**) for 120 min with or without 60 min pre-incubation with specific blockers (TAK242 (5 µM) for TLR4 and anti-human RAGE antibody (10 µg/mL) for RAGE). Then the TER was measured (**A, B and C**). All data are presented as mean ± s.d. of four independent experiments. Phosphorylation of p38 (p-p38) and ERK1/2 (p-ERK1/2) were also assessed by Western blotting (**D, E and F**). The ratio of immunointensity between the phosphorylation of p38 and ERK1/2 (p-p38 and p-ERK) and total p38 and ERK1/2 (p38 and ERK) were calculated (**D, E and F**). The results are expressed in mean ± s.d. from three independent experiments. ^**^P<0.01 *vs.* Control; ^#^P<0.05 *vs.* S100A8/S100A9; ^♣^P<0.05 *vs.* S100A8/S100A9+TAK242+anti-RAGE.

It is first approved that HUVECs used in this experiment constitutively expressed TLR4 and RAGE (Fig. S3). The preferences of S100A8 and S100A9 on TLR4 and RAGE were testified by using classical TLR-4 specific inhibitor TAK242 and RAGE blocking antibody, before S100A8 and S100A9 application. The inhibition of TLR4 with TAK242 (5 µM) attenuated S100A8-induced increase of monolayer hyper-permeability, and the addition of anti-RAGE (10 µg/mL) did not enhanced the TAK242-evoked decrease of S100A8-induced hyperpermeabiliby (Fig. 6A). TAK242 had no much influence on S100A9-induced endothelial barrier dysfunction. The blockage of RAGE with anti-RAGE abolished S100A9-evoked TER reduction (Fig. 6B). On the other hand, compared with TAK242 or anti-RAGE alone, the combination of TAK242 and anti-RAGE exerted greater effect on the attenuation of TER reduction in HUVECs exposed to S100A8/A9 (Fig. 6C). The statistical detail of these data is showed in Fig. S4.

Again, the dependencies of S100A8 on TLR4 and S100A9 on RAGE were further confirmed by the data that the usage of TLR4 inhibitor TAK242, but not antibody of RAGE, abolished S100A8-induecd p38 and ERK1/2 phosphorylation (Fig. 6D) while the application of RAGE antibody, but not TAK242, blocked S100A9-induced activations of p38 and ERK1/2 (Fig. 6E). Compared with TAK242 or anti-RAGE alone, the combination of TAK242 and anti-RAGE demonstrated greater inhibition of p38 and ERK phosphorylation in HUVECs exposed to S100A8/A9 (Fig. 6F).

### The calcium dependency in S100A8 and S100A9 evoked endothelial response

**Figure 7:**
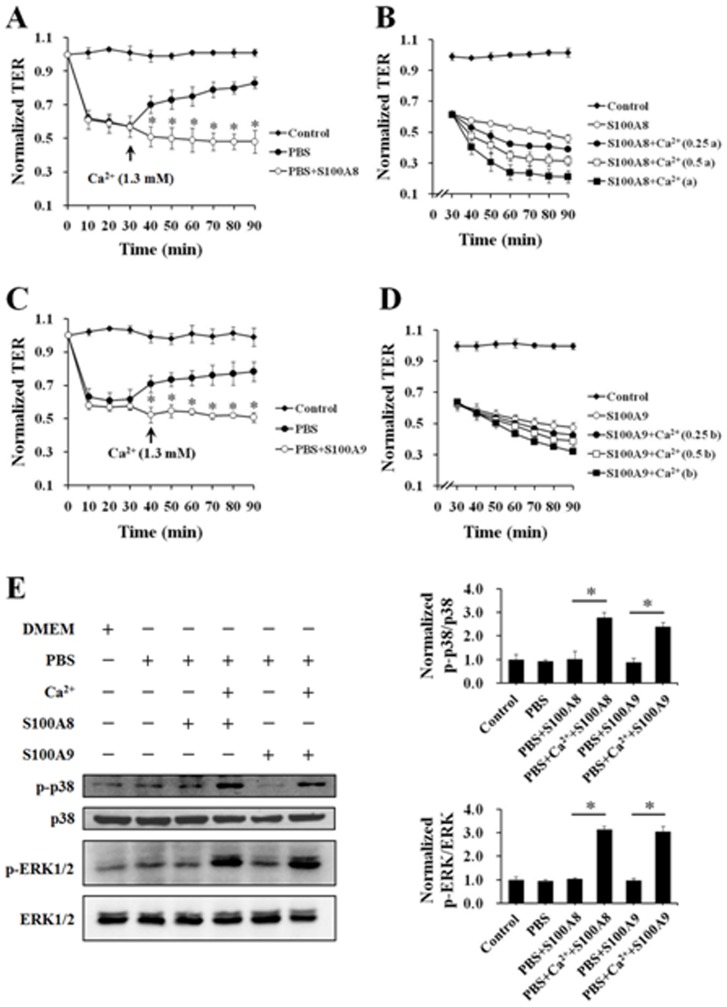
S100A8 and S100A9 increased HUVEC permeability in a Ca^2+^-dependent manner. (**A**) HUVECs were incubated with Ca^2+^-free medium (PBS) with and without S100A8 (2.0 µg/mL) for 90 min and at the 30th min 1.3 mM Ca^2+^ were added to the medium. TER was measured every 10 min. All data are presented as mean ± s.d. of four independent experiments. ^*^P<0.01 vs. PBS. (**B**) HUVECs were incubated with Ca^2+^ free medium (PBS) for 30 min. Then HUVECs were stimulated with S100A8 (2.0 µg/mL) which was pre-incubated with different concentration of Ca^2+^ (0, 0.25a, 0.5a and a. a = 0.37 µM. “a” represents the concentration of Ca^2+^ which can just saturate calcium binding sites of 2.0 µg/mL S100A8) for 60 min. TER was measured every 10 min. All data are presented as mean ± s.d. of four independent experiments. (**C**) HUVECs were incubated with Ca^2+^-free medium (PBS) with and without S100A9 (2.0 µg/mL) for 90 min and in the 30th min 1.3 mM Ca^2+^ were added to the medium. TER was measured every 10 min. All data are presented as mean ± s.d. of four independent experiments. ^*^P<0.01 vs. PBS. (**D**) HUVECs were incubated with Ca^2+^ free medium (PBS) for 30 min. Then HUVECs were stimulated with S100A9 (2.0 µg/mL) which was pre-incubated with different concentration of Ca^2+^ (0, 0.25b, 0.5b and b. b = 0.30 µM, “b” represents the concentration of Ca^2+^ which can just saturate calcium binding sites of 2.0 µg/mL S100A9) for 60 min. TER was measured every 10 min. All data are presented as mean ± s.d. of four independent experiments. (**E**) HUVECs were stimulated with S100A8 (2.0 µg/mL) and S100A9 (2.0 µg/mL) with or without Ca^2+^. Phosphorylation of p38 (p-p38) and ERK1/2 (p-ERK1/2) were assessed by Western blotting with specific antibodies as described in Materials and Methods. The ration of immunointensity between the phosphorylation of MAPKs (p-p38 and p-ERK) and total MAPKs (p38 and ERK) were calculated. The results are expressed in mean ± s.d. from three independent experiments. ^*^indicates P<0.01.

To decide the calcium dependency in S100A8 and S100A9 induced endothelial response, it is very important to consider the influence of extracellular calcium in intercellular junctions. Adherens junction molecule VE-cadherin relies on the presence of enough extracellular calcium to form an intercellular homodimers and play a pivotal role in endothelium integrity and in the control of vascular permeability [28]. Ca^2+^ here not only facilitates the formation of adherens junctions but also protects multicellular configuration by preventing the cadherins from hydrolysis. So in this study, the absence of calcium first caused a decline in TER due to the disruption of adherens junctions (Fig. 7). The effects of EGTA-induced depletion of extracellular calcium on endothelial permeability were also revealed, showing similar results with deprivation of calcium (Fig. S5). While the restore of proper calcium dose resumed the barrier function with a recovering TER, the appearances of S100A8 (Fig. 7A) or S100A9 (Fig. 7C) kept TER on decreasing. These results suggested that S100A8 and S100A9 abolished the re-annealing of previously open inter-endothelial junctions and disturbed the integrity of endothelial monolayer and those effects were calcium dependent.

As mentioned above, S100A8 and S100A9 have two calcium-binding sites (EF hands) per protein chain, so the dose necessity for saturate binding of calcium is countable according to the molecular weight of S100A8 (10.835 kDa) and S100A9 (13.242 kDa). In present study, the calcium dependency of S100A8 or S100A9 was further confirmed by adding different portion of saturate calcium concentration for calcium binding sites of 2.0 µg/mL S100A8 or S100A9. The results demonstrated that the presence of escalating dose of calcium enhanced the reduction of TER in S100A8- (Fig. 7B) or S100A9- (Fig. 7D) treated endothelial monolayer. The activation of p38 and ERK1/2 induced by S100A8 or S100A9 also showed the property of calcium-dependent. The absence of calcium abolished the phosphorylation of p38 and ERK1/2 under the treatment of S100A8 or S100A9 (Fig. 7E).

## Discussion

**Figure 8:**
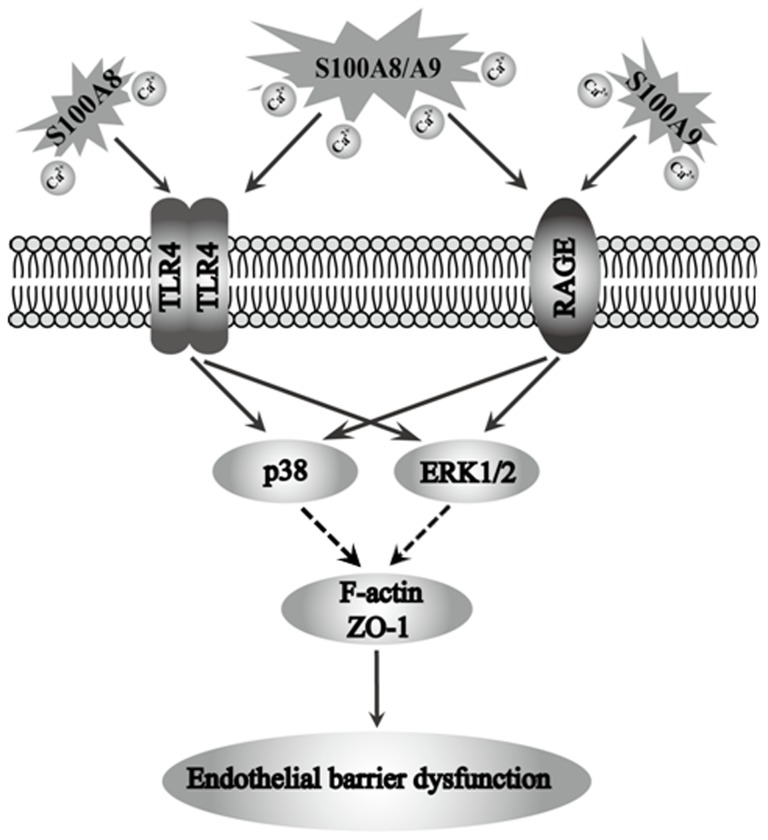
Schematic image of the signaling pathways of S100A8-, S100A9, and S100A8/A9-induced endothelial barrier dysfunction.

The present study demonstrated that S100A8 or S100A9 alone, as well as the heterodimers of S100A8/A9, induced the endothelial monolayer hyper-permeability in characteristic time patterns. These S100A8- and S100A9-induced endothelial responses were calcium-dependent. S100A8 seemed to mainly bind with TLR4, while S100A9 relied more on RAGE to introduce the cellular response. Although three subtypes of MAPKs, p38, ERK1/2, and JNK could be phosphorylated, only p38 and ERK1/2 were involvement in S100A8 and/or A9-induced monolayer hyper-permeability reaction in HUVECs (Fig. 8). This is the first study to separately reveal the instant effects and the underlying mechanisms of S100A8, S100A9, and S100A8/A9 on endothelial barrier function.

Viemann D. et al. has demonstrated the S100A8/A9-induced specific inflammatory response in endothelial cells on gene expression level [19]. Their report shows that S100A8/A9 induce a thrombogenic, inflammatory response in human microvascular endothelial cells by increasing the transcription of proinflammatory chemokines IL-8 and CXCL1, and adhesion molecules VCAM-1 and ICAM-1, and by decreasing the expression of intercellular junction protein cadherin. They further demonstrated that the long term impairment of endothelial integrity induced by S100A8/A9 is due to the induction of apoptosis [29]. The present study paid attention to the instant response of HUVEC monolayer permeability upon the challenge of S100A8, S100A9 and S100A8/A9, respectively. The data revealed that extracellular S100A8 evoked the disarrangement of F-actin and the disorganization of tight junction protein ZO-1, resulting in the increase of monolayer permeability in dose-dependent pattern in as early as 30 min. S100A9 and complex S100A8/A9 induced the disruption of endothelial barrier in more gradual manners, but the significance appeared in less than 1 h. These results suggest that S100A8, S100A9 and complex S100A8/A9 could educe the morphological reaction in endothelial cells, probably without the involvement of gene expression. This is the first study to compare the functional characteristics of homo S100A8, S100A9, and their hetero complex on HUVECs. It is also the first study focusing on the early influence of S100A8 and/or A9 on endothelial cells.

It has been well known that the major receptors for S100A8/A9 are TLR4 and RAGE. Endothelial cells express both TLR4 and RAGE and the expression are inducible under inflammatory stimulation [21], [22]. Robinson MJ et al. have provided evidences that S100A8, S100A9 and S100A8/A9 might have different dependencies on TLR4, RAGE, depends on the species and the cell types used [17]. The current study approved that these two receptors took part in the instant response in S100A8 and/or A9-challenged endothelial cells with different preference on TLR4 or RAGE. Vogl T et al. has revealed that S100A8 specifically interacts with TLR4 in phagocytes [25]. Bjork P et al.'s study showed that S100A8 has virtually no binding affinity to RAGE, but S100A9 had a high affinity to RAGE in the presence of Ca^2+^ and Zn^2+^ [30]. While carboxylated glycans is part of RAGE sub-structure, it is anti-S100A9, but not anti-S100A8 blocked neutrophil binding to immobilized carboxylated glycans [31], implying the ligand-receptor relation of S100A9 and RAGE. Consistently, the result in present study also suggested that S100A8 preferentially employed TLR4, S100A9 employed RAGE, to trigger the instant responses in HUVECs. But Bjork P et al's reports showed that S100A9 also had high affinity to TLR4/MD2 [30], and there are some other controversial data on the preference of S100A8 and/or A9 on TLR4 and RAGE [32]–[34]. It is necessary to further confirm the preference of S100A8 and/or A9 on different receptors by using knock-down cellular and animal models. In addition to TLR4 and RAGE, receptors for S100A8 and/or A9 include the scavenger receptor CD36 [35], glycosaminoglycans and heparin sulphate [17] in endothelial cells. This experiment could not rule out the involvement of these receptors in mediating the effects of S100A8 or S100A9 since TLR4 inhibitor and RAGE antibody only reduced, but did not eliminate S100A8 and/or A9-induced barrier dysfunction. Regarding the roles of heparin sulfate and CD36 in endothelial barrier function, it would be prudent to consider their effects in S100A8 and/or A9-induced response.

Different MAPK subtypes, p38, ERK1/2 and JNK, have distinct role in the regulation of cellular responses [36]. It has been shown in our previous study that p38 MAPK played a critical role in thermal injury by mediating formation of F-actin stress fiber and redistribution of ZO-1 in HUVECs. ERK1/2 was partially involved in burn serum-induced stress-fiber formation, while JNK had no influence on these endothelial morphological changes after thermal injury [37]. In this study, the results demonstrated that S100A8, S100A9 and S100A8/A9 triggered the phosphorylation of three MAPK subtypes via TLR4 or RAGE, but only p38 and ERK1/2 played a role in modulating S100A8 and/or A9-induced endothelial barrier dysfunction. These data are consistent with previous reports that p38 is the most active MAPK in affecting the endothelial morphological alteration [38], while ERK1/2 might also exert its effects on regulation of the endothelial barrier function [39], [40]. JNK might play its role in inducing the expression of endothelial adhesion molecules [41].

It is well known that MAPKs are the common downstream signal pathways both for TLR4 [42], [43] and RAGE [44], [45]. When this study only focused on these S100A8, A9-induced phosphorylation of p38, ERK1/2, and JNK, the results could not rule out the possibility that TLR4 and RAGE might also trigger some different downstream molecules to pass the signal of S100A8 and S100A9 for the regulation of endothelial barrier function.

It has to be very cautious when we evaluate the influence of calcium on S100A8, A9-induced endothelial barrier dysfunction since the existence of calcium is the fundamental condition for the integrity of intercellular adhesion junction. This dependency on calcium should be deducted at first. In this study, the deprivation of calcium caused a sharp decrease in TER due to the lack of calcium in intercellular adhesion junctional connection. This result is consistent with the report from Legrand P et al. that the cadherin multimer dissociates into monomers in the absence of Ca^2+^ [46]. The full recovery of extracellular calcium concentration restored the barrier integrity in PBS-treated cells, but not in plus S100A8 or S100A9-treated cells, suggesting that S100A8 or S100A9 hampered the restoration of barrier function and even worsened the disruption of endothelial barrier in the presence of calcium. The dependency on calcium was revealed again by the results that S100A8- or S100A9-induced lowering of TER was enhanced as the calcium concentration was gradually elevated, demonstrating that the calcium-dependent formation of S100A8 or S100A9 homodimer causes the disruption of endothelial barrier function.

In summary, this present study demonstrated that S100A8 or S100A9 homodimers, as well as S100A8/A9 heterodimers, can damage the endothelial barrier function by activating MAPK signal pathway through receptors TLR4 and RAGE. The calcium dependent formation of S100A8 and S100A9 dimmers might be the prerequisite for this S100A8/A9-induced endothelial functional alteration. The most important finding of this study is the revelation of the preference of S100A8 on TLR4 and S100A9 on RAGE. The disadvantage of present study is that there was no analysis on the formation of monomers, homodimers for S100A8 and S100A9. The formation of heterodimers of S100A8/A9 was also not detected.

## Supporting Information

Figure S1 **S100A8, S100A9 and S100A8/A9 induced a concentration- and time-dependent increase in HUVEC permeability.** Filter-grown HUVEC monolayers were stimulated for 120 min with S100A8 (0, 0.5, 1.0, 1.5, 2.0, 4.0) (**A**), S100A9 (0, 0.5, 1.0, 1.5, 2.0, 4.0) (**B**) and S100A8/A9 (0, 0.5, 1.0, 1.5, 2.0, 4.0) (**C**) respectively. The TER was measured every 10 min. All data are presented as mean ± s.d. of four independent experiments. ^*^P<0.05 *vs.* Control.
(TIF)

Figure S2 **S100A8, S100A9, or S100A8/S100A9 were heat-inactivated at 80°C for 30 minutes.** HUVEC monolayer was stimulated for 120 min with inactivated S100A8 (2.0 µg/ml), S100A9 (2.0 µg/ml) or, S100A8/S100A9 (2.0 µg/ml) respectively. TER was then measured. All data are presented as mean ± SD of four independent experiments (A). Phosphorylation of ERK1/2 (P-ERK1/2) was assessed by Western blotting. The ratio of immunointensity between the phosphorylation of ERK (P-ERK) and total ERK was calculated from three independent experiments (B). ^*^indicated *P* > 0.05.
(TIF)

Figure S3 **The expression of TLR4 and RAGE in HUVECs used in this experiment.** Cells were maintained in DMEM/F12 containing 10% FBS and grown to 90% confluence. HUVECs were starved of serum for 12 hours then lysised with SDS loading buffer. The expression of TLR4 and RAGE were assessed by Western blotting with primary antibodies for TLR4 (1∶1000, Cat. AF1478) and RAGE (2 ug/ml, Cat. MAB11451) (R&D Systems, Minneapolis, MN).
(TIF)

Figure S4 **The effects of blocking TLR4 and RAGE on S100A8, S100A9 and S100A8/A9 stimulation of HUVECs.** HUVECs were stimulated with S100A8 (2.0 µg/mL) (**A**), S100A9 (2.0 µg/mL) (**B**) and S100A8/A9 (2.0 µg/mL) (**C**) for 120 min with or without 60 min pre-incubation with specific blockers (TAK242 for TLR4 and anti-human RAGE antibody for RAGE). Then the TER was measured. ^*^P<0.05 vs. Control, ^♠^P<0.05 *vs.* S100A8, ^♣^P<0.05 *vs.* S100A9, ^&^P<0.05 *vs.* S100A8/A9, ^♦^P<0.05 *vs.* S100A8/A9+TAK242+anti-RAGE.
(TIF)

Figure S5 **The effects of EGTA-induced depletion of extracellular calcium on endothelial permeability were also revealed, showing similar results with deprivation of calcium.**
(TIF)
