# Supplementary figures and images for: Functional Characterization of S100A8 and S100A9 in Altering Monolayer Permeability of Human Umbilical Endothelial Cells
Source: PLoS One. 2014 Mar 3;9(3):e90472. doi: 10.1371/journal.pone.0090472 (PMC3940892; doi:10.1371/journal.pone.0090472)

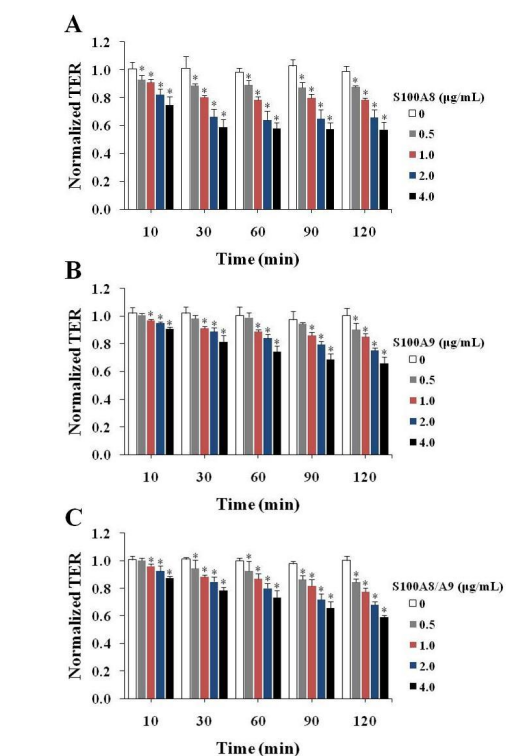

Supplement: Figure S1 — S100A8, S100A9 and S100A8/A9 induced a concentration- and time-dependent increase in HUVEC permeability. Filter-grown HUVEC monolayers were stimulated for 120 min with S100A8 (0, 0.5, 1.0, 1.5, 2.0, 4.0) (A), S100A9 (0, 0.5, 1.0, 1.5, 2.0, 4.0) (B) and S100A8/A9 (0, 0.5, 1.0, 1.5, 2.0, 4.0) (C) respectively. The TER was measured every 10 min. All data are presented as mean ± s.d. of four independent experiments. *P<0.05 vs. Control. (TIF) [file pone.0090472.s001.tif]

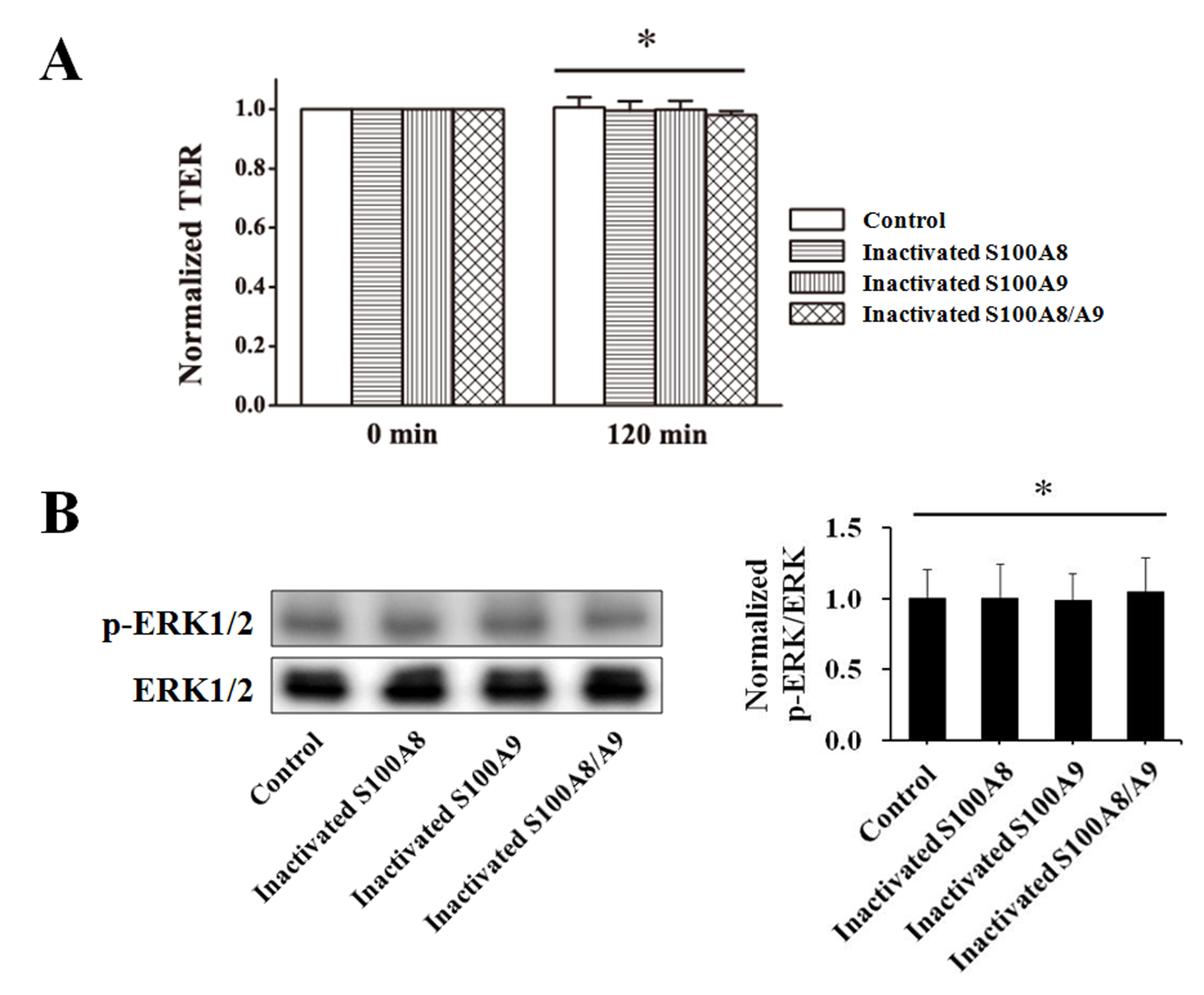

Supplement: Figure S2 — S100A8, S100A9, or S100A8/S100A9 were heat-inactivated at 80°C for 30 minutes. HUVEC monolayer was stimulated for 120 min with inactivated S100A8 (2.0 µg/ml), S100A9 (2.0 µg/ml) or, S100A8/S100A9 (2.0 µg/ml) respectively. TER was then measured. All data are presented as mean ± SD of four independent experiments (A). Phosphorylation of ERK1/2 (P-ERK1/2) was assessed by Western blotting. The ratio of immunointensity between the phosphorylation of ERK (P-ERK) and total ERK was calculated from three independent experiments (B). *indicated P > 0.05. (TIF) [file pone.0090472.s002.tif]

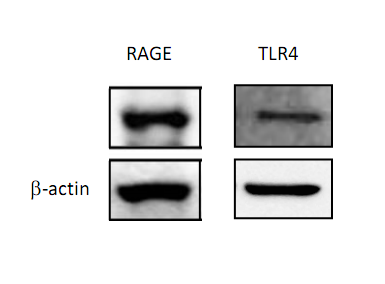

Supplement: Figure S3 — The expression of TLR4 and RAGE in HUVECs used in this experiment. Cells were maintained in DMEM/F12 containing 10% FBS and grown to 90% confluence. HUVECs were starved of serum for 12 hours then lysised with SDS loading buffer. The expression of TLR4 and RAGE were assessed by Western blotting with primary antibodies for TLR4 (1∶1000, Cat. AF1478) and RAGE (2 ug/ml, Cat. MAB11451) (R&D Systems, Minneapolis, MN). (TIF) [file pone.0090472.s003.tif]

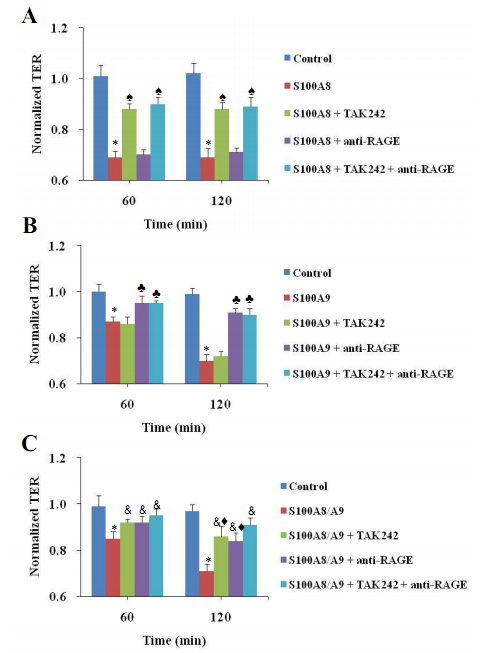

Supplement: Figure S4 — The effects of blocking TLR4 and RAGE on S100A8, S100A9 and S100A8/A9 stimulation of HUVECs. HUVECs were stimulated with S100A8 (2.0 µg/mL) (A), S100A9 (2.0 µg/mL) (B) and S100A8/A9 (2.0 µg/mL) (C) for 120 min with or without 60 min pre-incubation with specific blockers (TAK242 for TLR4 and anti-human RAGE antibody for RAGE). Then the TER was measured. *P<0.05 vs. Control, ♠P<0.05 vs. S100A8, ♣P<0.05 vs. S100A9, &P<0.05 vs. S100A8/A9, ♦P<0.05 vs. S100A8/A9+TAK242+anti-RAGE. (TIF) [file pone.0090472.s004.tif]

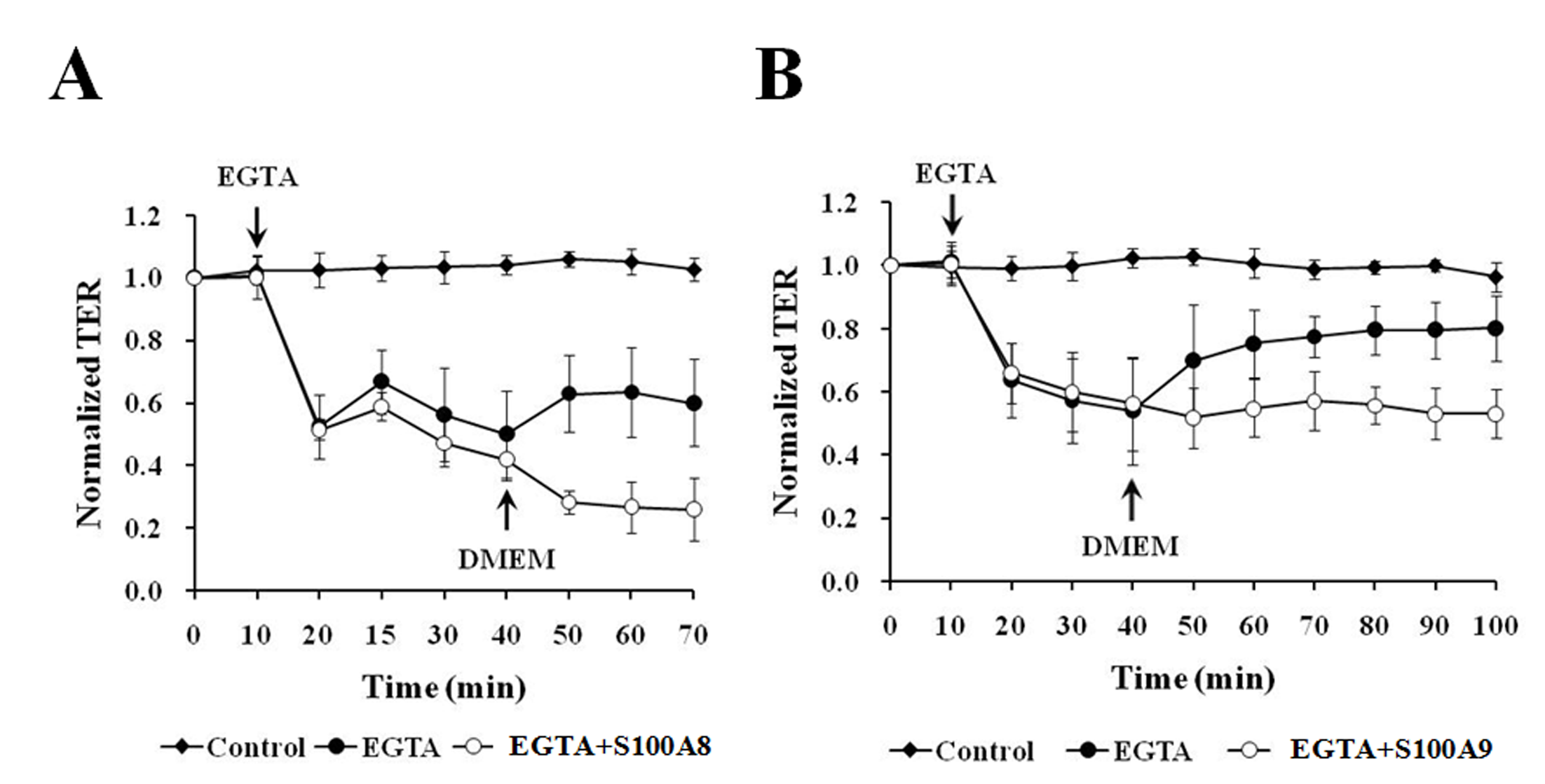

Supplement: Figure S5 — The effects of EGTA-induced depletion of extracellular calcium on endothelial permeability were also revealed, showing similar results with deprivation of calcium. (TIF) [file pone.0090472.s005.tif]
